# Complement-ARIE: Catalyzing the development and adoption of new approach methodologies

**DOI:** 10.1016/j.namjnl.2025.100026

**Published:** 2025-05-17

**Authors:** Kristifor Sunderic, Andrew M. Wright, Nicole Kleinstreuer, Victoria Ledbetter, Katelynn A. Milora, Christine Happel, Daniel Shaughnessy, Chariz P. Johnstone, Daniel Anthony Casco, Nikeya Macioce, Jacqui Marzec, Kristin Kano, Margaret J. Ochocinska, Danilo A. Tagle

**Affiliations:** a National Center for Advancing Translational Sciences, National Institutes of Health, Bethesda, MD 20892, USA; b Office of the Director, National Institutes of Health, Bethesda, MD 20892, USA; c National Institute of Environmental Health Science, National Institutes of Health, Bethesda, MD 20892, USA

**Keywords:** New approach methodologies, Novel alternative methods, NAMS, Microphysiological systems, MPS, National institutes of health, NIH, Complement Animal Research In Experimentation, Complement-ARIE, In vitro, In chemico, In silico

## Abstract

New approach methodologies (NAMs) are proving to be invaluable tools in basic and clinical research to better understand human health and disease, elucidate mechanisms, and study the efficacy and toxicity of novel therapeutics that can improve upon and complement animal-based methodologies. Over the last 15 years, the National Institutes of Health (NIH) has increased investment in NAMs, including in chemico, in silico, and in vitro approaches, such as cell-free assay systems, digital twins, and microphysiological systems (MPS). To further catalyze and innovate the use of NAMs, the NIH Common Fund has initiated a new program, Complement Animal Research In Experimentation (Complement-ARIE), which aims to pioneer the development, standardization, validation, and regulatory use of combinatorial NAMs that will more accurately model human biology and disease states. This program specifically aims to 1) better model human health and disease differences in outcomes across populations, 2) develop NAMs that can provide insights into specific biological processes or disease states, 3) validate mature NAMs to support regulatory use and standardization, and 4) complement traditional animal models to make research more efficient and effective. To inform the implementation of Complement-ARIE, the NIH organized strategic planning activities including an inter-agency retreat, public listening sessions, scientific literature landscape analysis, and a challenge prize. Here we discuss the goals and findings of these activities, and how these results helped identify and address scientific and operational opportunities and roadblocks for implementation of the program towards broader acceptance and impact of NAMs. We anticipate these new methods and approaches could transform the way we do basic, translational, and clinical sciences.

## 1 Introduction

Scientific breakthroughs are often propelled by the introduction of new technologies that can transform how scientists study health and disease, and the 21st century has been a time of accelerated technological advancement. Increasing use of new and improved biomedical technologies, such as gene editing, artificial intelligence (AI), human induced pluripotent stem cells (iPSCs), and microphysiological systems (MPS) or tissue chips, are fundamentally changing the way science is performed. These approaches serve to equip researchers with new methods that offer unique precision and, for specific types of studies, can potentially reduce reliance on animal model systems and increase human relevance. These New Approach Methodologies (NAMs), sometimes referred to as “Novel Alternative Methods or Non-Animal Models,” provide a complementary approach to traditional models while offering tremendous promise for enhancing understanding of the human system and for more effectively treating human conditions (Ingber, 2022; Kleinstreuer et al., 2014; Kleinstreuer and Holmes, 2021).

Preclinical research serves as a crucial gateway to drug development, bridging fundamental scientific discovery with tangible patient outcomes, yet often fails to yield safe and effective medicines. <10 % of drugs entering clinical trials result in approved medicines, in part due to species-specific differences (Sun et al., 2022; Wong et al., 2019). About 56 % of drug candidates fail due to lack of efficacy in humans and 28 % fail due to toxic effects in humans (Arrowsmith and Miller, 2013). Pharmacogenomics also differ between humans and animal models, playing a role in poor translation rates. For example, many animal models are inbred lines that poorly account for the genetic variability of humans. Furthermore, the accumulation of environmental exposures, stressors, and life experiences in various human populations contributes uniquely to how diseases manifest in individuals and how they respond to treatments. Animal models alone do not accurately simulate the complexities of disease presentations and potential treatment responses in humans (Zeggini, 2019). As such, pivoting from the use of animal models to rely more on human-biology based systems can lead to results with improved predictive safety and efficacy profiles that can guide more reliable development of treatments. For the time being, animal models still play a critical role in pre-clinical biomedical research, but development of other approaches that complement or potentially replace animal models is sorely needed.

### 1.1 Applications of new approach methodologies

NAMs, which broadly span in vitro, in chemico, and in silico approaches (Table 1.), have proven to be valuable tools in basic, clinical, and toxicology research, and have been used to study the efficacy and toxicity of novel therapeutics.

**Table 1:** NAMs defined (FDA Modernization Act 2.0).

**In silico**: Experiments performed by computing platforms or custom hardware, encompassing mathematical modeling and simulation, machine learning, and other computational techniques.
**In chemico**: Experiments performed on biological molecules, such as proteins and DNA, outside of cells, which may be used to study how these molecules interact with each other and with drugs.
**In vitro**: Experiments performed on cells outside of the body, including various types of MPS, cell, organoid, and tissue culture techniques.

Modern in vitro NAMs employ 2D and 3D cell cultures often in conjunction with bioprinting and MPS to recapitulate organ and tissue human physiology. Examples include patient-derived cells to create organoids for ovarian cancer and development of prostate organoids for preclinical cancer research (Buskin et al., 2023; Chan et al., 2023). Related non-cancer NAMs include induced pluripotent stem cell (iPSC)-derived cardiomyocyte “heart-on-a-chip” systems, hepatocyte microfluidic “lab-on-a-chip” platforms, and ocular organoids for drug development and disease research (Bai and Wang, 2020; Criscione et al., 2023; Di, 2023).

In chemico NAMs are often cell-free biochemical assays used for biomarker structure and identity analysis. For example, a proteomic NAM approach has helped to identify prognostic, predictive, and therapeutic biomarkers for various sarcoma subtypes (Connolly et al., 2023). A second example is the AR2 assay, which uses a luciferase enzyme fused to the human androgen receptor to assess androgen receptor homodimerization and can be combined with human cytochrome P450 (CYP) metabolism to capture the bioactivity of test compounds (Brown et al., 2023). A third example is a histone deacetylase 1 (HDAC1) intracellular imaging system that utilizes a supramolecular tandem assay and fluorescent reporter pair to identify HDAC1 binding and screen HDAC1 inhibitors (Li et al., 2023).

In silico NAMs are mostly computational approaches focused on AI and machine learning (ML), molecular simulation, digital twins, and predictive models for absorption, distribution, metabolism, and excretion. Large language models have also been used to generate predictions from biomedical literature (Zagirova et al., 2023). *In silico* technologies are revolutionizing toxicology (Kleinstreuer and Hartung, 2024) and accelerating and optimizing product design, as well as patient diagnostic and treatment strategies (Kleinstreuer and Kreuzer, 2024).

Applications of NAMs extend outside disease modeling and drug testing and have been used to assess environmental exposure risk factors. For example, human primary cell-based systems combined with computational models have been used to assess the inhalation risk of the pesticide chlorothalonil (Clippinger et al., 2018). NAMs can thus enable regulatory agencies like the Environmental Protection Agency (EPA) to reduce dependency on vertebrate animal testing to efficiently assess health hazards in the environment. Furthermore, with the recent adoption of the Food and Drug Administration (FDA) Modernization Act 2.0 (FDA Modernization Act 2.0) and the April 2025 announcement of the FDA Roadmap to Reducing Animal Testing in Preclinical Safety Studies (Roadmap to reducing animal testing in preclinical safety studies), there is significant progress being made towards incorporating NAMs as nonclinical tests to demonstrate safety and efficacy of therapeutics. The legislation permits the use of specific alternatives to animal testing, including different NAMs, for assessing drug safety and effectiveness during the preclinical phase (Michelson, September 2023). This has paved the way for NAMs to bolster the preclinical data pipeline, aiming to reduce dependence on animal models that have frequently resulted in therapeutic dead ends, and presents an important opportunity to improve preclinical-to-clinical trial pipelines for new therapeutics.

### 1.2 NIH complement-Arie program

In May 2022, in response to a US Congressional request, the NIH reported to Congress on prior investments in NAMs. Following the report, an Advisory Committee to the Director (ACD) Working Group was convened, comprised of experts across multiple disciplines and sectors to assist the NIH in prioritizing the development of NAMs with the highest potential for catalyzing biomedical research (ACD Working group on catalyzing the development and use of novel alternative methods to advance biomedical research). The ACD Working Group was asked to identify the landscape of NAMs currently being deployed, assess their strengths and limitations, and characterize the types of research in which they hold the most promise of complementing and/or replacing animal studies. The ACD Working Group met with developers and users of NAMs to explore not only the current context of use, but also opportunities for future development and deployment. Seven key recommendations (Table 2.) were identified by the working group.

**Table 2:** Summary of Recommendations to catalyze the development and use of NAMs (Catalyzing the development and use of novel alternative methods).

i. Prioritize the development and use of combinatorial NAMs
ii. Establish resources, infrastructure, and collaborations to promote the use of interoperable, reliable, and well curated/high quality datasets produced from research using NAMs
iii. Promote effective dissemination and interconnection of NAMs technologies
iv. Invest in comprehensive training to bolster continuous advances in NAMs development and use
v. Facilitate in multidisciplinary teams with expertise across technologies and the lifecycle of NAMs development and use
vi. Promote social responsibility in both the creation and deployment of NAMs across the research lifecycle
vii. Support and maintain coordinated infrastructure to catalyze effective and responsible NAMs development and use

The ensuant NIH-wide Complement-ARIE program aims to address many of these recommendations and advance the development, standardization, validation, and use of new methods and approaches that will more accurately model human biology and complement, or in some cases, replace traditional animal models, transforming the way we do basic, translational, and clinical science. Successful deployment of NAMs, whether for conducting basic research, uncovering disease mechanisms, or translating knowledge into products or practice, relies on bringing together many disciplines, technologies, data, and areas of expertise. The Complement-ARIE working group (WG) engaged in robust strategic planning activities and scientific community outreach focused on developing a unifying vision to catalyze ongoing NAM efforts that develop, standardize, validate, and use NAMs, and identifying opportunities for innovation and coordination. Feedback from the scientific community was solicited through the Complement-ARIE Ideation Challenge to propel the development and refinement of human-based NAMs (Complement-ARIE Challenge prize winner summaries). These strategic planning activities yielded several challenges and areas of focus that could result in significant advancement of NAMs.

## 2 Strategic planning

### 2.1 Listening sessions

#### 2.1.1 Background

The Complement-ARIE WG hosted three public listening sessions to gather unique perspectives, insights, and recommendations from key representatives in multiple sectors on current opportunities and roadblocks in NAMs development. These included industry and academic researchers, non-governmental organizations (NGOs), U.S. government scientists and policymakers, and international partners. Approximately 1100 people registered, and over 550 participated across all sessions.

The feedback provided, both in-person and through written submissions, centered around the following topics:

##### 2.1.1.1 Translating animal models to human conditions

Participants discussed the limitations of translating findings from animal studies to humans for diseases involving complex biological interactions. Autoimmune diseases, neurodevelopmental and neurodegenerative diseases, cognitive and psychiatric disorders, pregnancy and reproductive complications, cancer, respiratory diseases, and metabolic disorders were all discussed as particularly challenging domains.

Also discussed was how differences in physiology, immune response, genetic variability, xenobiotic metabolism, toxicokinetics, pharmacokinetics, and lifespan can limit the applicability of animal models to human health and disease. For example, short-lived animal models can be insufficient in replicating human aging in chronic diseases, which also limits how well animal models can capture how diseases develop and manifest in humans. Additionally, relative genetic similarity in animal models means they cannot fully represent the genetic variability of human populations and that research pathways for rare diseases are often limited.

Participants noted animal models seldom capture the dynamic social and environmental factors that affect human health. Lab animals are often housed in sterile and homogenous environments that do not mirror the complex environments humans experience and subjective measures like pain management, psychological disorders, or disability recovery are difficult to model and interpret in animals in ways that accurately approximate human pathology.

Despite ethical standards for animal research, there can be deleterious impacts on research workforce. Representatives from NGOs emphasized the negative toll working with laboratory animals has on the mental health and well-being of biomedical workers, particularly when the animals are in pain. Finally, it was noted that animals are often expensive to obtain and maintain in laboratory settings and are not rapidly scalable to biomedical research needs for drug development, lead optimization, or toxicological screening.

At the conclusion of the discussion, participants from NGOs, the U.S. government, and international partners recommended NIH conduct a systematic assessment to identify animal models with poor translatability to inform future funding decisions for NAMs.

##### 2.1.1.2 Current limitations of NAMs

Like animal models, existing NAMs have limitations in replicating population variability, dynamic environments, and complex human biology and disease. Technology constraints, lack of standardization in NAM protocols, and availability of harmonized datasets are also challenges.

Participants addressed how NAMs are currently deficient in modeling age-dependent diseases and the progression of chronic disorders over time. In vitro NAMs are typically developed using cell lines or young cells (e.g., iPSCs) which support modeling of early-stage human development but do not mimic mature cells in the human body. NAMs that model neurological disorders and behavioral endpoints are also inadequate, and there is a lack of long-term data to model the chronic and cumulative effects of aging on cells in vitro.

Participants discussed gaps in the ability of NAMs to accurately mimic diseases involving communication between multiple cell types, tissues, or organ systems in the body. For example, in silico NAMs are currently constrained by a lack of high-quality training data and insufficiencies in the high-performance computing technology required to model complex diseases. However, a systems approach may not always be required, and considering the context of use can help connectivity between tissues as a limitation.

Also addressed was how NAM platforms do not currently represent dynamic, human-relevant exposures, including changes in the internal environment (e.g., hormone fluctuations) and the synergistic effects of multiple chemical exposures and social stressors in humans. NAMs are also limited in their ability to model the metabolism and pharmacokinetics of drugs. Current limitations for in vitro to in vivo extrapolation analyses must be resolved to improve the confidence in NAMs results. High cost and low throughput are also barriers to the predictive capability and widespread adoption of current NAMs. Additionally, current cell lines are not derived from a sufficiently varied set of donors so NAMs do not suitably represent human population variability. Participants called for a focus on community engagement to improve donor variability and addressed limited access to data and tissue banks as constraints for developing NAMs.

Generally, participants spoke about how a lack of standardization regarding data collection and annotations, NAMs development, and validation endpoints complicates inter-laboratory collaboration and acceptance by end users. Data standards are needed to facilitate the sharing and harmonization of NAMs data. As recommended by the ACD Working Group, a database is needed to catalog existing NAMs that includes the context of use, results, validation status, and best practices for each NAM. The database can build upon or coordinate with existing NAMs databases as well. Participants provided examples of existing databases as a reference, including the Microphysiological Systems Database (now EveAnalytics) (TSAR - tracking system for alternative methods towards regulatory acceptance) and the European Commission Tracking System for Alternative Methods Towards Regulatory Acceptance (TSAR - tracking system for alternative methods towards regulatory acceptance), which track NAMs and their validation status. Such a resource is being planned and supported by the NIH via the National Interagency Center for the Evaluation of Alternative Toxicological Methods (NICEATM) (NICEATM: alternative methods), and will be called the Collection of Alternative Methods for Regulatory Acceptance (CAMERA) (Collection of alternative methods for regulatory application).

Finally, representatives from federal agencies expressed that there are currently no guidelines for how to properly cite NAMs in publications, often making it difficult to discover what work is being done with NAMs in what areas.

##### 2.1.1.3 The potential for NAM applications

Despite current limitations, participants expressed enthusiasm that NAMs have a wide-ranging potential beyond animal models to accelerate biomedical research discoveries. NAMs could leverage existing datasets from large human population studies and cell banks of patient-derived iPSCs to improve our understanding of the mechanisms driving the pathophysiology of human diseases including initiation, progression, prevention, and treatment. If catalyzed and developed, NAMs could allow for improved modeling of myriad health conditions, including rare diseases, neurological disorders, cancers, wound healing, and chronic illnesses across the lifespan and across populations. The use of digital twins and virtual patient models were noted by representatives from the U.S. government, international groups, and NGOs as promising approaches that could achieve some of these advancements.

Participants posited that in silico and in vitro approaches could facilitate the development of safer and more effective drugs. NAMs could be used to predict adverse clinical trial outcomes, rapidly screen for potential drug interactions, and generate more accurate pharmacogenomic profiles. Patient-derived iPSCs can enable a precision medicine approach, thus providing patient-specific personalized disease prevention and treatment options.

Additionally, NAMs could be used to conduct large-scale, high throughput testing of environmental agents to facilitate complex risk assessments, such as for large groups of chemicals like per- and polyfluoroalkyl substances (PFAS) and assess potential health impact of chronic agent exposures. Participants expressed that NAMs have the potential to facilitate modeling and simulations for rapid response situations such as infectious disease outbreaks, food contamination, chemical spills, or disasters.

Participants also noted that integrating data across various NAM platforms, anchoring NAMs in the whole-body context, and incorporating genetic and other types of variabilities into NAMs will be critical to advancing the field.

##### 2.1.1.4 Building confidence and validation of NAMs

Barriers to widespread acceptance of NAMs include uncertainty around validation requirements and processes, lack of familiarity with NAMs data affecting confidence in their use by regulators and end-users, and lack of support for validation studies. Increasing scientific confidence in NAMs is a pressing issue, and participants in all sessions spoke about the need for frameworks to standardize how NAMs are developed, how data is collected and annotated, and how validation and qualification studies need to be conducted (e.g. via widespread adoption of guidance released by the Interagency Coordinating Committee on Validation of Alternative Methods (ICCVAM)) (Validation, qualification, and regulatory acceptance of new approach methodologies).

To build confidence in NAMs, participants called for a government-led effort to inform and train all partners on how to interpret NAMs data and methods’ usefulness and limitations, focusing on context of use and flexible approaches to validation (particularly when direct comparison to animal data is not possible or appropriate).

Participants from all sessions agreed that creating opportunities for regulators, developers, and end-users to collaborate early and often is necessary to facilitate NAMs development, validation, and adoption. Representatives from academia and NGOs noted the need to set reasonable regulatory expectations for NAMs as not all areas of applications will need regulatory approval. Some participants also noted the importance of validating NAMs against existing in vivo data to facilitate regulatory acceptance and uptake by end users. In support of this, it is critical that NAMs demonstrate relevance to human biology such as by reflecting key events along adverse outcome pathways.

Participants re-emphasized the need for additional infrastructure to support NAMs validation, including tissue banks, data-sharing infrastructure, and a registry of NAM methods and data that have been accepted for regulatory purposes and their context of use (e.g. CAMERA).

##### 2.1.1.5 Enabling widespread adoption of NAMs

Achieving widespread adoption of NAMs will require collaboration among industry, academia, government, and regulatory agencies. These groups should work together to establish common goals, achieve standardization, and share data and resources. Cooperation between NAMs developers and end users is also critical. Representatives from academia and industry noted that public-private partnerships for data sharing will be key to accelerating NAMs development and reducing end users’ uncertainty around NAMs. Interdisciplinary collaborations should bring together experts in materials science, computational modeling and analyses, toxicology, clinical research, and drug discovery.

The bias towards animal-based methods as the status quo was another barrier to the widespread adoption of NAMs that was discussed. Participants recommended an educational initiative aimed at grant reviewers, journal publishers, regulators, and end users to help demonstrate NAMs relevance to human biology and build confidence in the methods.

Training scientists, particularly early-stage investigators, is vital to increasing adoption of NAMs and continued growth in the field. Training should focus on NAMs development, use, limitations, opportunities, and oversight, as well as integration into multidisciplinary research for continuing development and use across multiple sectors. Participants recommended institutional training programs to train researchers while promoting collaboration.

Access to costly NAMs infrastructure and disparate data was identified as a major barrier to uptake. Participants suggested creating a centralized tissue bank and data repository and leveraging existing initiatives, like the NIH All of Us Program (All of us Research Program), to help researchers access the resources needed to transition from animal studies to NAMs research. Participants also recommended the establishment of a center program to provide researchers access to shared core facilities and the interdisciplinary expertise necessary to stimulate NAMs adoption and progress.

### 2.2 Interagency retreat

#### 2.2.1 Background

Representatives from multiple federal agencies, including NIH, FDA, EPA, NSF, ARPA-H, BARDA, VA, DARPA, NIST, NASA,^2^ and the inter-agency ICCVAM committee, convened for a two-day retreat hosted by the NIH to discuss goals and priorities for advancing NAMs. Through presentations, panel discussions, and breakout sessions, agency representatives highlighted various NAMs programs currently underway in their respective fields and organizations. These included MPS or organs on chips (OoCs), virtual models, 3D cell cultures, organoids, stem cell models, AI/ML, computational systems methods, and more. The retreat participants identified scientific areas that could benefit from NAMs, including cancer research, clinical trials, chronic diseases, aging, adaptive immunity, neuroscience, and precision health.

#### 2.2.2 Priorities and challenges

The retreat underscored several priorities that will enable broader acceptance and impact of NAMs. Agency representatives expressed that clear standards are needed across all aspects of NAMs, from data generation methodologies to validation frameworks to reproducibility efforts that could facilitate regulatory approval and use of NAMs. The participants also indicated that regulatory agencies should require NAM data adhere to FAIR (findable, accessible, interoperable, and reusable) principles.

There was consensus from participants that NAM data, particularly from validation studies, should be publicly available, as well as associated regulatory guidance on consideration or acceptance of NAMs. Information should be included from validated NAMs and de-identified research, as well as aggregated data from industry partners, such as from U.S. FDA drug submissions. Such a database could employ AI to extract and compile data into a searchable database that would facilitate the development of new NAMs and highlight gaps where NAMs are unavailable, as well as areas where traditional models are not predictive of human responses. In addition, the creation of harmonized data-sharing and knowledge graph networks, such as NSF’s Prototype Open Knowledge Network (Proto-OKN) currently in development, would provide a wealth of data to inform NAMs in the future.

Agency partners re-established validation of NAMs as a significant challenge. They also underscored the need for collaborative and pre-competitive environments, fit-for-purpose validation frameworks, and facilitating interplay between researchers and experts who focus on validation.

Transparent communication is needed to develop public understanding of and trust in NAMs. Retreat attendees agreed that agencies should collaborate on simple, cohesive, and coordinated messaging to share with public audiences. According to agency representatives, the most effective, resource-efficient near-term use of NAMs is to complement traditional models. Although creating NAMs to replace traditional models might be applicable in some situations, the goal is to create NAMs that fill knowledge gaps that current models cannot address. Communication with the public should emphasize this message.

#### 2.2.3 Insights

Several high-level themes emerged from the discussions on regulatory, operational, and scientific needs for NAMs.

##### 2.2.3.1 Regulatory needs

Databases of NAMs, including accepted and validated NAMs, could inform widespread NAM uptake into regulation as well as NAM development by helping identify where traditional models are not sufficient or not available. Participants expressed uncertainty about which NAMs are available for use or validated, and which can be built upon or used for different purposes. Further, the amount of data available for NAM development and validation must be increased and be publicly available, harmonizable, and reusable.

Participants stressed that context of use is critical when considering regulatory acceptance. Regulators not only look at the reproducibility and quality of NAMs but also consider the cost and efficiency of NAMs versus current models, e.g. via value of information analysis.

##### 2.2.3.2 Scientific opportunities

Participants cited short-term opportunities for NAMs such as:

■ Using AI to extract and compile publicly available data from FDA drug submissions into a searchable database of animal-based, in vitro, and human-based information for building and assessing models.
■ Validating NAMs by testing compounds with known toxicity profiles in multi-organ chips with human versus animal-derived cells and then establishing confidence by matching animal data with NAM data.
■ Incorporating more cell types and biological complexity into NAMs to better model physiology and disease states.
■ Using AI and computational models to learn from failed clinical trials to improve predictive ability of models to capture genetic, environmental, and societal factors affecting health.

Proposed long-term opportunities included:

■ Developing models to assess chronic exposures over time, as well as models for aging.
■ NAMs that represent disease susceptibility and mechanisms across varied populations.
■ Integrating data across NAMs, animals, and humans to build better predictive models.
■ NAMs for precision medicine for individual risk assessment and personalized health.

### 2.3 Landscape analysis

To ensure that the Complement-ARIE program focused on the areas of science with the greatest need, and which present the best opportunities for human-based model development, a landscape analysis of the biomedical research literature was undertaken to collect information on ongoing efforts in the NAMs space.

The landscape analysis was intended to provide a foundation on which to better define the scope of Complement-ARIE and inform coordination with existing programs. It included a survey of in vitro, in chemico, and in silico approaches that have the potential to improve understanding of human health and disease mechanisms, reduce reliance on animal models, and make use of animals more efficient. The landscape analysis was summarized in a report published online (Complement animal research In experimentation (Complement-ARIE), landscape analysis Report 2024: new approach methodologies in biomedical research) and presented via an interactive Tableau data visualization dashboard.

Of the responses received, results suggested that cardiomyocytes, neuronal and endothelial cell types, and liver, bone, breast, lung, and skin tissues are extensively represented among in vitro NAMs. A particular focus on cancer, diabetes, Alzheimer’s and Parkinson’s diseases was identified, although there were few articles that included the application of NAMs in translation for clinical relevance, and there was a general lack of incorporation of immune and microvascular components of these disease. This highlights less established cell types/tissues and diseases areas that should be targeted for further development.

There was more modest representation of in chemico methods, which predominantly comprised biochemical and structure/identity analysis techniques. These methods have the capacity to efficiently and sensitively screen compounds of interest and thus could be considered in future research efforts to design combinatorial NAMs approaches. Given the rapid growth of computational approaches, it is not surprising that AI and ML represent the majority of identified in silico NAMs. There is little debate of the prospective power of such approaches in biomedical research, limited only by the quality and quantity of input data with which AI models are initially developed and trained.

Future efforts should harness the data generated by NAMs; therefore, an essential Complement-ARIE program priority is the development of a readily accessible data repository. Another priority is the establishment of metadata reporting guidelines or standards; the landscape analysis demonstrated that databases incorporating curation/harmonization steps and detailed reporting criteria or forms tended to adhere more to FAIR principles. Regardless of the category of method considered, economic and workforce considerations, ethical considerations, regulatory use, and population variability were not adequately addressed, highlighting key areas where additional research efforts should be directed. Given their potential throughput, human relevance, and opportunity for representing individual heterogeneity, it was concluded that continued evaluation of the utility of NAMs in addressing these research questions should be part of the Complement-ARIE Program.

### 2.4 Common themes

The listening session, interagency retreat, and landscape analysis provided overlapping insights, demonstrating their importance to the future of the Complement-ARIE program. These were:

1) **Interoperable databases**: For Complement-ARIE to catalyze the development and maturation of NAMs, the data, methods, and regulatory experience garnered by the program must be centrally accessible, adhere to FAIR standards, and be publicly available.
2) **Standardization and validation:** A lack of clear standards is pervasive among NAMs. Complement-ARIE must develop collaborative infrastructure to facilitate validation of NAMs and support the harmonization of NAMs standards as solutions mature. Adoption of NAMs is often hampered by animal models being considered the international gold standard. Validation and confidence-building that emphasizes human biological and mechanistic relevance of NAMs will increase acceptance across a range of communities.
3) **Engagement and training with end-users**: Effective and efficient adoption of NAMs requires a trained workforce and clear messaging around NAMs relationships with animal models. Communication strategies should build upon structured validation efforts to emphasize the robustness, relevance, and replicability of NAMs.

### 2.5 Prize challenge

#### 2.5.1 Background

Complement-ARIE launched a crowdsourced prize competition to source innovative ideas for NAMs development that may benefit from further investment. Submissions requirements included formation of a multidisciplinary team and NAMs solutions that were both novel and feasible across competition areas of in silico, in vitro, in chemico, and combinatorial methods. Twenty Complement-ARIE Challenge prize winners shared the total prize purse of $1,000,000, with each winning team receiving $50,000 for their innovative solutions.

The winning proposals addressed gap areas identified during strategic planning efforts and highlighted strategies to develop integrated solutions for NAMs that could more effectively model human-based end points. A majority of submissions were related to complex in vitro human-derived cell or tissue-based models. Highly rated submissions focused on integrated or combinatorial solutions as well as multi-disciplinary approaches that had more likelihood for long-term impact in the field and were aligned with the prospective goals of the Complement-ARIE Program.

The prize challenge drew a range of submissions from industry groups and academia, often combining researchers from different fields such as bioengineering, chemistry, data science, and clinical medicine. These groups represented non-traditional NIH applicants and indicated the broader outreach that a prize competition could achieve. These varied backgrounds underscored the interdisciplinary nature of challenge prize submissions, and the potential for Complement-ARIE to spur innovative breakthroughs in biomedical research and multidisciplinary collaboration. Concepts from the winning entries are being incorporated into Complement-ARIE program planning, and a full list of winners has been published (Complement-ARIE Challenge prize winner summaries).

## 3 Complement-ARIE program implementation

**Fig. 1:**
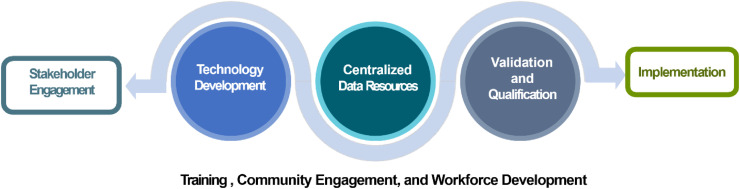
Key components of the Complement-ARIE program including technology development, centralized data resources, validation and qualification, along with key training and dissemination activities to provide a fluid conduit from engagement to implementation.

Strategic planning activities informed the goals Complement-ARIE research program and have helped define its three key components: technology development, centralized data resources, and validation and qualification (Fig. 1.). These components will exist cooperatively under the umbrella of the Complement-ARIE consortium, harnessing stakeholder engagement to bring together the expertise of interdisciplinary experts and facilitate coordinated implementation.

### 3.1 Technology development centers

Technology Development Centers (TDCs) will be NAMs development centers focused on high priority areas of greatest need, with emphasis on biological complexity, throughput, innovative combinatorial approaches, capture of population variability and susceptibility, and resource and data sharing according to FAIR principles. High priority scientific needs identified during strategic planning activities include, but are not limited to: chronicity (i.e. across the lifespan), neuroscience (e.g. neurodegenerative disease models, neuropsychiatry, ophthalmology, behavioral research), personalized health (e.g. human-specific models to address the rapid growth in biological therapeutics, including monoclonal antibodies, human proteins, oligonucleotides, gene editing, and cell therapies, and incorporating population variability and sex as a biological variable), and cross-disease pathogenesis (e.g. developmental, metabolic, immune, reproductive health). TDCs will foster workforce development and capacity building through built-in training and outreach components to sustainably bolster advances in NAMs development, dissemination, deployment, and adoption. TDCs will also support community engagement to enhance the impact and adoption of NAMs in a human relevant framework.

Combinatorial NAMs, or strategies integrating in vitro, in chemico, and in silico strategies for specific applications, are a major focus of Complement-ARIE and should be realized through TDCs as final deliverables. Ideally, TDCs will generate mature combinatorial NAMs with clinical utility for defined use cases that will progress to validation and qualification in partnership with regulatory authorities and regulated industry.

### 3.2 NAMs data hub and coordinating center

The NAMs Data Hub and Coordinating Center (NDHCC) will integrate data structures via a central data hub. This will help to develop and apply standards for data reporting and model credibility and improve FAIR adherence by NAM-relevant data, including data interoperability and data-reuse strategies. The NDHCC will create a searchable NAMs repository, develop tools for data analytics, and lower barriers for sharing NAMs-related data with researchers and the wider community.

### 3.3 Validation and qualification network

Successful adoption of NAMs for biomedical, preclinical, and regulatory use requires that all sectors with appropriate skills and resources work together in innovative and collaborative ways to accelerate NAMs validation. Accordingly, the NIH in collaboration with the Foundation for the NIH (FNIH) is in the process of establishing a Validation and Qualification Network (VQN) (FNIH - validation & Qualification Network Design phase) through a Public-Private Partnership (PPP) involving scientists at multiple levels of government (including funding agencies and regulators), industry, nongovernmental organizations, and academic institutions. The shared expertise the VQN will uniquely catalyze the development, standardization, and validation of human-based NAMs.

The VQN will establish a process for evaluating NAMs and channeling use cases through its framework in a pre-competitive environment, paving the way for broader adoption and application by industry and biomedical researchers globally. The goals of the VQN include:

• Accelerating deployment and implementation of NAMs in both research and regulatory contexts.
• Developing standardized reporting and common data elements for preclinical, clinical, and safety performance.
• Developing standardized frameworks for NAMs regulatory submissions.

After the VQN PPP is established, it will transition to an initial Design Phase followed by an Implementation Phase. The Design Phase will include a series of workshops with parties in NAMs-related fields to determine governance, scope, and priorities for NAMs validation and qualification efforts. The Implementation Phase will support the generation of data packages and regulatory submissions consistent with established validation/qualification frameworks. The VQN will also collaborate with the other components of the Complement-ARIE consortium, namely the TDC and NDHCC, and will work closely with ICCVAM, which will be essential to coordinate across multiple federal partners. By working with collaborators aimed at commercial market use and surveillance, the VQN will help to streamline submission of new NAMs or NAMs-related data to regulatory authorities. Complement-ARIE is specifically focused on the development and validation of novel combinatorial NAMs and will build upon existing U.S. and international efforts to ultimately provide more cost-effective, rapid, human-relevant NAMs for drug discovery, chemical safety testing, and wider biomedical research approaches to bring NAMs to market.

## 4 Conclusion

The new NIH Common Fund Complement-ARIE Program engaged in robust strategic planning activities and outreach focused on crafting a unifying vision to develop, standardize, validate, and propagate NAMs. By including the subject matter expertise of partners across industry, academia, NGOs, and other domestic/international entities, the program identified critical opportunities and high-priority areas for NAMs innovation and coordination. Engagement with a range of partners helped identify areas of science with the greatest need for NAMs development. A landscape analysis collected and analyzed ongoing efforts in the NAMs space and identified ongoing challenges and opportunities. A prize competition provided information about where NAMs innovation could be effectively pursued and what novel NAMs would benefit from further investment.

These activities informed the strategic substance and scope of the Complement-ARIE Program to maximize its efficacy and impact to advance our understanding of human health and disease. Complement-ARIE aims to use this strong platform to catalyze partnerships across the biological, toxicological, and biomedical research enterprise.

Whether it be to expand our research toolkit or enable new inquiries into challenging diseases, we firmly believe NAMs will transform our collective capabilities to promote health.

Disclaimer: The views and opinions expressed in this manuscript are those of the authors only and do not necessarily represent the views, official policy or position of the U.S. Department of Health and Human Services or any of its affiliated institutions or agencies.

## Financial Support

None.

## CRediT authorship contribution statement

**Kristifor Sunderic:** Writing – original draft. **Andrew M. Wright:** Writing – review & editing. **Nicole Kleinstreuer:** Conceptualization, Writing – review & editing. **Victoria Ledbetter:** Writing – original draft. **Katelynn A. Milora:** Writing – original draft. **Christine Happel:** Writing – original draft. **Daniel Shaughnessy:** Writing – review & editing. **Chariz P. Johnstone:** Writing – original draft, Writing – review & editing. **Daniel Anthony Casco:** Writing – original draft. **Nikeya Macioce:** Writing – original draft. **Jacqui Marzec:** Writing – original draft. **Kristin Kano:** Writing – original draft. **Margaret J. Ochocinska:** Writing – original draft, Writing – review & editing. **Danilo A. Tagle:** Conceptualization, Methodology, Project administration, Supervision, Writing – original draft, Writing – review & editing.

## Declaration of competing interest

The authors declare the following financial interests/personal relationships which may be considered as potential competing interests: Danilo A. Tagle reports administrative support was provided by 10.13039/100000002National Institutes of Health. Danilo A. Tagle reports a relationship with 10.13039/100000002National Institutes of Health that includes: employment. If there are other authors, they declare that they have no known competing financial interests or personal relationships that could have appeared to influence the work reported in this paper.

## Data Availability

No data was used for the research described in the article.

## Footnotes

2 FDA – Food and Drug Administration; EPA – Environmental Protection Agency; NSF – National Science Foundation; ARPA-H – Advanced Research Projects Agency for Health; BARDA – Biomedical Advanced Research and Development Authority; VA – Department of Veterans Affairs; DARPA – Defense Advanced Research Projects Agency; NIST – National Institute of Standards and Technology; NASA – National Aeronautics and Space Administration

## References

[bib0001] "ACD Working group on catalyzing the development and use of novel alternative methods to advance biomedical research," [Online]. Available: https://acd.od.nih.gov/working-groups/novel-alternatives.html.

[bib0002] "All of us Research Program," [Online]. Available: https://allofus.nih.gov/.

[bib0003] Arrowsmith J., Miller P. (2013). Phase II and Phase III attrition rates 2011–2012. Nat. Rev. Drug Discov..

[bib0004] Bai J., Wang C. (2020). Organoids and microphysiological systems: new tools for ophthalmic drug discovery. Front Pharmacol..

[bib0005] Brown E., Hallinger D., Simmons S. (2023). High-throughput AR dimerization assay identifies androgen disrupting chemicals and metabolites. Front. Toxocol..

[bib0006] Buskin A., Scott E., Nelson R., Gaughan L., Robson C., Heer R., Hepburn A. (2023). Engineering prostate cancer in vitro: what does it take?. Oncogene.

[bib0007] "Catalyzing the development and use of novel alternative methods," National Institutes of Health, Bethesda, Maryland, 2023.

[bib0008] Chan W., Mo X., Ip P., Tse K. (2023). Patient-derived organoid culture in epithelial ovarian cancers—techniques, applications, and future perspectives. Cancer Med..

[bib0009] Clippinger A., Allen D., Jarabek A., Corvaro M., Gaça M., Gehen S., Hotchkiss J., Bartels M., BéruBé K., Wilson D., Indans I., Vinken M. (2018). Alternative approaches for acute inhalation toxicity testing to address global regulatory and non-regulatory data requirements: an international workshop report. Toxicol. Vitro.

[bib0010] "Collection of alternative methods for regulatory application," [Online]. Available: https://ntp.niehs.nih.gov/sites/default/files/2024-10/4-3_Kleinstreuer_SACATM2024_508.pdf.

[bib0011] "Complement animal research In experimentation (Complement-ARIE), landscape analysis Report 2024: new approach methodologies in biomedical research," National Institutes of Health, 2024.

[bib0012] "Complement-ARIE Challenge prize winner summaries," [Online]. Available: https://commonfund.nih.gov/complementarie/challengewinnersummaries.

[bib0013] "Complement-ARIE Challenge prize winner summaries," [Online]. Available: https://commonfund.nih.gov/complementarie/challengewinnersummaries.

[bib0014] Connolly E., Grimison P., Horvath L., Robinson P., Reddel R. (2023). Quantitative proteomic studies addressing unmet clinical needs in sarcoma. Front Oncol..

[bib0015] Criscione J., Rezaei Z., C.M H.C., Murphy S., Shin S., Kim D. (2023). Heart-on-a-chip platforms and biosensor integration for disease modeling and phenotypic drug screening. Biosens. Bioelectron..

[bib0016] Di L. (2023). Recent advances in measurement of metabolic clearance, metabolite profile and reaction phenotyping of low clearance compounds. Expert. Opin. Drug Discov..

[bib0017] "FDA Modernization Act 2.0," [Online]. Available: https://www.congress.gov/bill/117th-congress/senate-bill/5002.

[bib0018] "FNIH - validation & qualification network design phase," [Online]. Available: https://fnih.org/our-programs/validation-qualification-network-design-phase/.

[bib0019] Ingber D. (2022). Human organs-on-chips for disease modelling, drug development and personalized medicine. Nat. Rev. Genet..

[bib0020] Kleinstreuer N., Hartung T. (2024). Artificial intelligence (AI)-it's the end of the tox as we know it (and I feel fine). Arch Toxicol..

[bib0021] Kleinstreuer N., Holmes A. (2021). Harnessing the power of microphysiological systems for COVID-19 research. Drug Discov. Today.

[bib0022] N. Kleinstreuer and S. Kreuzer, "In Silico Technologies: a strategic imperative for accelerating breakthroughs and market leadership for FDA-regulated products," Reagan-Udall Found., 2024.

[bib0023] Kleinstreuer N.C., Yang J., Berg E.L., Knudsen T.B., Richard A.M., Martin M.T., Reif D.M., Judson R.S., Polokoff M., Dix D.J., Kavlock R.J., Houck K.A. (2014). Phenotypic screening of the ToxCast chemical library to classify toxic and therapeutic mechanisms. Nat. Biotechnol..

[bib0024] Li M., Yu H., Li Y., Li X., Huang S., Liu X., Weng G., Xu L., Hou T., Guo D., Wang Y. (2023). Rational design of supramolecular self-assembly sensor for living cell imaging of HDAC1 and its application in high-throughput screening. Biosens. Bioelectron..

[bib0025] Michelson G. (1 September 2023). A new path to new drugs: finding alternatives to animal testing. Science.

[bib0026] "NICEATM: alternative methods," [Online]. Available: https://ntp.niehs.nih.gov/whatwestudy/niceatm.

[bib0027] "Roadmap to reducing animal testing in preclinical safety studies," [Online]. Available: https://www.fda.gov/media/186092/download. 10.1002/cpdd.70046 41766281

[bib0028] Sun D., Gao W., Hu H., Zhou S. (2022). Why 90 % of clinical drug development fails and how to improve it?. Acta Pharm. Sin. B.

[bib0029] "TSAR - tracking system for alternative methods towards regulatory acceptance," [Online]. Available: https://tsar.jrc.ec.europa.eu/.

[bib0030] "Validation, qualification, and regulatory acceptance of new approach methodologies," [Online]. Available: https://ntp.niehs.nih.gov/sites/default/files/2024-03/VWG_Report_27Feb2024_FD_508.pdf. 40418713

[bib0031] Wong C.H., Siah K.W., Lo A.W. (2019). Estimation of clinical trial success rates and related parameters. Biostatistics.

[bib0032] Zagirova D., Pushkov S., Leung G., Liu B., Urban A., Sidorenko D., Kalashnikov A., Kozlova E., Naumov V., Pun F., Ozerov I., Aliper A., Zhavoronkov A. (2023). Biomedical generative pre-trained based transformer language model for age-related disease target discovery. Aging.

[bib0033] Zeggini E. (2019). Translational genomics and precision medicine: moving from the lab to the clinic. Science.

